# Surface Proximity Effect, Imprint Memory of Ferroelectric Twins, and Tweed in the Paraelectric Phase of BaTiO_3_

**DOI:** 10.1038/s41598-018-31930-4

**Published:** 2018-09-12

**Authors:** C. Mathieu, C. Lubin, G. Le Doueff, M. Cattelan, P. Gemeiner, B. Dkhil, E. K. H. Salje, N. Barrett

**Affiliations:** 1 SPEC, CEA, CNRS, Université Paris-Saclay, CEA Saclay, 91191 Gif sur Yvette cedex, France; 2 School of Chemistry, University of Bristol, Cantocks Close, Bristol, BS8 1TS United Kingdom; 3 Laboratoire Structures, Propriétés et Modélisation des Solides, CentraleSuplec, CNRS-UMR8580, Université Paris-Saclay, 91190 Gif-sur-Yvette, France; 4 Department of Earth Sciences, University of Cambridge, Downing Street, Cambridge, CB2 3EQ United Kingdom

## Abstract

We have used energy-filtered photoemission electron microscopy (PEEM) at the photoemission threshold to carry out a microscopic scale characterization of the surface charge and domain structure of the (001) surface in BaTiO_3_. Signatures of ferroelectric and ferroelastic domains, and tweed, dominate the surface structure of BaTiO_3_ at room temperature. The surface ferroic signatures are maintained on heating to temperature (~550 K), well above the transition temperature (393 K). This surface proximity effect provides the mechanism for memory of the bulk ferroelectric domain arrangement up to 150 K above T_C_ and thus can be considered as a robust fingerprint of the ferroelectric state near the surface. Self-reversal of polarization is observed for the tweed below T_C_ and for the surface domains above T_C_. Annealing at higher temperature triggers the dynamic tweed which in turn allows a full reorganization of the ferroic domain configuration.

## Introduction

The paraelectric (PE) to ferroelectric (FE) phase transition in the model ferroelectric BaTiO_3_ (BTO) is mainly abrupt and of first order from the high cubic symmetry (Pm3m) to the lower tetragonal symmetry (P4mm) structure at 393 K^1^. Defects and high domain wall concentrations can soften the transition. The permittivity and the domain size depends on the grain size of the ceramic making the transition diffuse^2^. Within the framework of the order-disorder model the transition may also soften slightly^3^. BaTiO_3_ is also ferroelastic, characterized by 180° and 90° domains, with c/c as well as a/a and a/c domain walls^4–9^. Microscopic fluctuations in the strain order parameter, called tweed, are observed at temperatures above 393 K and can give rise to polarity^10,11^. Local FE distortions in the cubic phase have been highlighted by quasielastic neutron scattering^12,13^, X-ray absorption fine structure^14^ and by resonant ultrasonic spectroscopy^15^. Polar precursor ordering prior to a stepwise transition at the Curie temperature in BaTiO_3_ was also detected by resonant piezoelectric spectroscopy^16^. Dul’kin *et al*. showed the presence of a relaxor-like behavior with polar nanoregions in the same phase^17^. Tweed has been predicted for any ferroic material and is commonly observed both in displacive^18^ and order/disorder systems^19^.

Twinning and tweeding are hence well established as bulk ferroic phenomena but little is known of these effects at surfaces^20^. The intersection of twin boundaries with the surface have been shown to lead to singularities of the surface strain which reflect directly the thickness of the twin boundaries^21^ while equivalent investigations of tweed structures are still in their infancy^10^.

The ferroic surface patterns are expected to transform at much higher temperatures than the bulk phase transitions as electrostatic and elastic boundary conditions significantly alter the thermodynamic stability fields^22^. Indeed, phase transitions at the surface may occur at much higher temperatures than in the bulk and domain-like ordering have been seen in ferroic materials at the surface well above the transition point of the bulk^23,24^. Morozovska *et al*. have shown how surface ionic charge can couple with bulk ferroelectric states to create specific ferroionic domain patterns at the surface of thin films. These patterns may persist well above the bulk Curie temperature (T_C_)^25^. Höfer *et al*. showed that the signature of these surface charges can persist up to 510 K. Rumpling, reconstruction and relaxation, associated with the FE state, can be considerably altered at the surface^26,27^. However, although enhanced surface tetragonality due to the outward movement of oxygen ions may favor polarity^24^ it does not necessarily explain the persistence of different polarization states. The temperature reproducibility of these states above T_C_ is key to understanding their origin. Surface and interface properties are crucial for applications, particularly in nanoelectronics where they may dominate the bulk ferroelectric behavior^28–32^ Yet none of the surface studies reported the domain pattern when cooling back down to room temperature.

We use photoemission electron microscopy (PEEM) with *in-situ* temperature control to investigate the surface polarity of ferroelectric BaTiO_3_ by probing the local potential modulations at the microscopic scale^33^. PEEM provides parallel imaging in photoemission using electron lenses with a spatial resolution of ~50 nm. Domains with different FE polarization present different surface charge, which shifts the electronic levels and hence the work function of the emitted electrons. The photoelectrons have a small inelastic mean free path (from a few angstroms to a few nanometers, depending on the electron kinetic energy) making the technique inherently surface sensitive.

The present study analyzes local surface charges of a BaTiO_3_ (001) single crystal through the FE to PE phase transition. We demonstrate the persistence of FE-domain patterns at the surface up to 550 K, far above the bulk phase transition as measured by Raman spectroscopy. In addition, quasi-static surface tweed also survives up to the same temperature. Self-reversal of polarization is observed for the tweed below T_C_ and for the surface domains above T_C_. The FE domain structure and hence FE memory is lost when the tweed becomes dynamic after annealing at 975 K while it conserves the fingerprint of the initial state up to 550 K, i.e. up to 150 K above T_C_.

## Results

### Domain and tweed imaging

**Figure 1:**
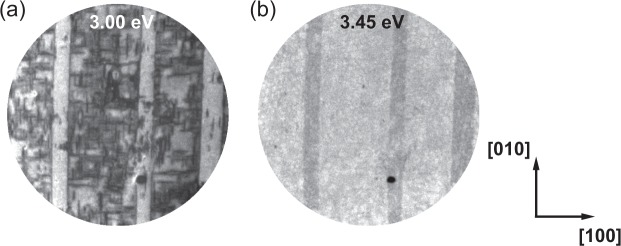
PEEM images at E-E_F_ = 3.00 and 4.45 eV at 300 K, showing contrast inversion in the intensity of the broad vertical stripes due to the difference in the local photoemission threshold. Within the stripes finer, tweed structure is observable with different intensity. The field of view is 67 *μ*m and images are recorded at 300 K.

Figure 1 shows typical PEEM images taken at two different values of E-E_F_: 3.00 and 3.45 eV at 300 K. We observe arrays of broad, parallel dark and bright vertical stripes corresponding to different ferroelectric domains. The contrast inversion between 3.00 and 3.45 eV for the stripe domains is a clear signature of at least two distinct photoemission threshold values, related to different surface polarization charge. Inside these stripes, fine tweed structure is observed with a distinctive intensity level suggesting a third polarization at the surface.

**Figure 2:**
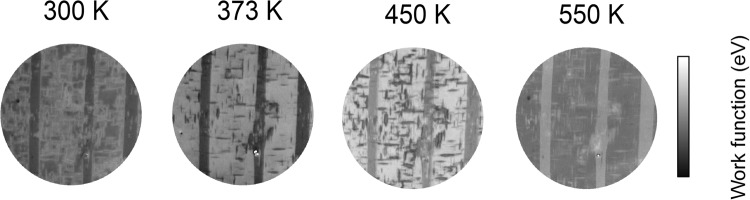
Work function maps obtained from image series below T_C_ at 300, 373, 450 and 550 K. The field of view is 67 *μ*m and the work function range spans in each case 0.5 eV. Dark grey corresponds to P^↓^, intermediate grey to P^in^ and light grey to P^↑^ polarization directions.

Image series have been recorded at four temperatures: 300, 373, 450 and 550 K and the local work function distribution calculated using the pixel by pixel analysis described in Methods. The resulting work function maps are shown in Fig. 2).

### Room temperature work function map

The map at room temperature of the initial state (Fig. 2) shows two characteristic, superimposed patterns, namely wide vertical stripe domains and fine-scale tweed. The orientation of the spontaneous polarization is revealed by the grayscale. The narrower vertical stripes have a lower work function, while the broader vertical stripes have an intermediate shade of gray on the work function color scale. The thinner stripes contain polarity pointing from the surface to the bulk (P^↓^). The intermediate grey is typical for polar vectors parallel to the surface (P^in^), i.e. zero polarity perpendicular to the surface. These patterns, with long straight walls along high symmetry directions, are typical for the intersection of 90° ferroelectric stripe domains with the surface^4,34^. The domain configuration is hence related to 90° walls inclined by ~45° to the surface. The tweed structure has the highest work function, representing positive surface charge, i.e. with polarization vector pointing from the surface into vacuum (P^↑^).

Thus, the broad vertical stripe patterns represent ferroelectric domains with uniform polarization but are related to the finer, tweed like arrays. The tweed arrays indicate that the surface contains large fluctuations of the polarization pointing towards the surface (dark), parallel to the surface (intermediate grey) and away from the surface (light grey). They form criss-crossed patterns typically observed in transmission electron microscopy and in computer simulations of tweed. These patterns possess no clearly defined length scale but are rather invariant over a large range of length scales.

### Work function maps as a function of temperature

The sample was then gradually heated from room temperature to 550 K. During heating, image series were recorded at several temperatures. The Curie temperature of the first order phase transition was identified from the sudden displacement of the sample in the PEEM image (see Supplementary Materials S1).

The work function maps at room temperature, 373, 450 and 550 K show remarkable similarities (Fig. 2). The most important observation is that there is no break in the overall pattern of tweed and domains at the bulk phase transition temperature T_C_ = 393 K. Both the wider vertical stripe domains and the tweed are still visible in the paraelectric phase. However, an important change in contrast between the tweed and the broader, vertical stripe domains is observed at 373 K. Whereas at room temperature the tweed work function is higher than that of the surrounding domain surface, at 373 K the tweed work function is less than that of the surrounding domain, i.e. surface charge has changed sign and therefore polarization reversal has taken place in the tweed.

There are also shifts in the absolute values of work function with temperature. This is due to the reduced sample charging with temperature and represents an offset but does not affect the work function contrast between different surface polarizations. It is completely reversible, the work function map obtained at room temperature after heating at 550 K is identical to that acquired before annealing (Fig. 4, discussed below).

The stepwise bulk transition is well seen using Raman spectroscopy that probes the BaTiO_3_ to a thickness of 500–600 nm (see Supplementary Materials S2). We can contrast, therefore, the bulk transition as identified by Raman with the behavior of the surface layer: the bulk does indeed assume the cubic symmetry while the surface layer remains tetragonal and conserves the FE pattern formation similar to that in the bulk tetragonal phase. Even at 550 K the surface conserves the fingerprint of the bulk FE tetragonal phase. The low symmetry of the surface layer compared with the cubic bulk structure requires an intermediate layer where two-phase coexistence occurs. If such layers are narrow, they constitute invariant planes^35,36^. The domain configuration near the invariant plane is well studied in alloys^37^ and consists of arrays of needle domains with tips oriented towards the cubic phase^38–41^.

**Figure 3:**
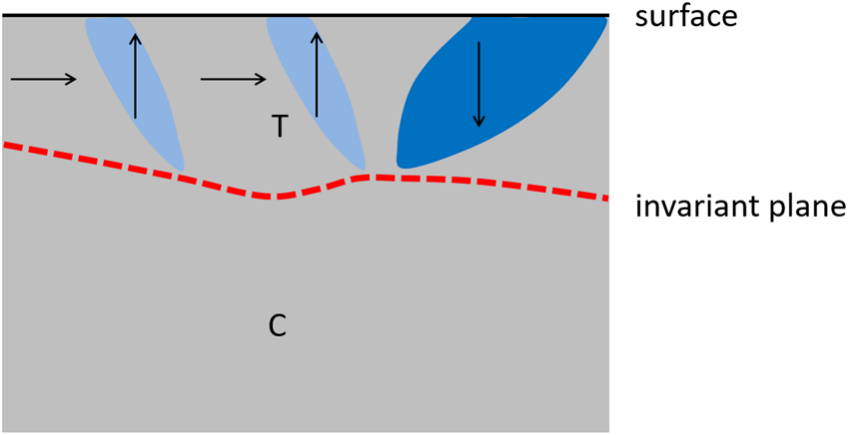
The sketch is a side view of the sample polarization state, for temperature above T_C_. The surface is represented by a dark line and the invariant plane by a red dashed line. The bulk is in the cubic (C) phase, while the surface remain in the tetragonal (T) phase. The polarization directions are represented by dark arrows.

We envisage a similar scenario here. The stripe domains penetrate the bulk at low temperatures and the observed patterns in PEEM reflect their intersections with the surface. As shown by the Raman spectroscopy, heating the sample transforms the bulk to a domain-free cubic state above T_C_ while the surface sensitive PEEM demonstrates that a few nanometer surface region maintains a ferroelectric domain structure. In this region, the remaining stripe domains retract into needle domains. Whereas 90° ferroelectric twin walls should be straight and for BaTiO_3_ lie in {110} planes, curvature is intrinsic to the needle domain wall^42^. Needle curvature is also observed experimentally^43^. The sketch in Fig. 3 represents schematically short, curved needle domains in the surface tetragonal layer with the bulk transformed into cubic phase. The depolarizing field, E_dep_, is a signature of polarization bound charges and its magnitude is inversely proportional to the sample thickness. When bulk FE domains disappear, the nanometer thin surface region naturally experiences a stronger E_dep_, which was previously compensated by the bulk domain structure. This finally leads to self-reversal of the polarization in the surface layer. Self-switching is well-known in thin films, where it is also termed backswitching^44^.

This effect is observed in Fig. 2 where the narrower vertical stripes (downwards polarization) observed at 300, 373 and 450 K take on a higher work function than the broader stripes when the temperature is increased to 550 K, i.e. they become upwards polarized. A video showing the rapid self-reversal is available with the Supplementary Materials (S3). In order to minimize the suddenly strong E_dep_ the surface breaks down into smaller domains. The needles become therefore increasingly shorter and finally constitute dagger domains pinned to the surface^45–47^, as represented by the sketch in Fig. 3. Short daggers contain fewer charges and thereby reduce the depolarization charge at the surface.

### High temperature annealing

**Figure 4:**
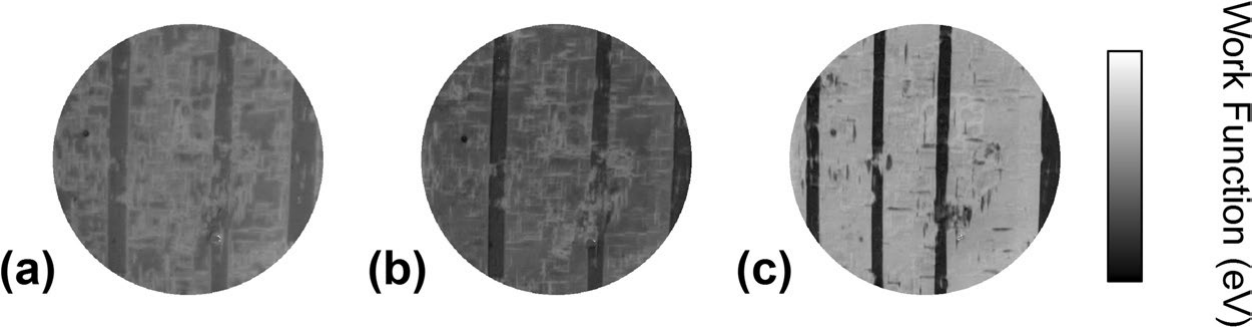
Surface potential maps of (**a**) initial surface, (**b**) after heating at 550 K and (**c**) after heating at 975 K. The maps are generated from image series recorded as a function of electron kinetic energy at room temperature. The field of view is 67 *μ*m in diameter and in each case the work function variation spans 0.5 eV.

Annealing the sample to higher temperatures leads to changes in the domain order at the surface as seen after quenching. In Fig. 4, the initial room temperature pattern (left) is compared with the patterns of the samples quenched to room temperature after annealing. Quenching from 550 K leads to essentially the same domain pattern, although some of the finer details of the tweed patterns have changed. The position of the stripe domains are exactly the same as before the thermal treatment and the tweed can be considered as quasi-static. This can be contrasted with the quench from 975 K where the domain pattern has changed. The dark stripe domains are narrower and have moved. The tweed, however, is the same as the initial pattern but part of the tweed kept the high temperature self reversal, corresponding to upwards polarization, whereas the other part has reverted to the original downwards polarization. All detailed positions are altered and the overall pattern appears slightly coarser. We observe also larger patches of horizontal orientations of the spontaneous polarization. We can assume, therefore, that heating to 975 K erases all memories of the initial domain configuration, possibly leading to a more uniform surface relaxation of the cubic phase.

## Discussion

### Switching kinetics

We have shown that the stepwise bulk phase transition in BaTiO_3_ does not occur at T_C_ in the surface layer. Here, the surface domain pattern imprinted by the ferroelectric bulk phase, namely stripe domains, coincides with a tweed-like pattern, in which the polarization vector can change between the three symmetry allowed directions. The persistence of domain-like ordering above the transition point has already been observed in PEEM^24^, and scanning surface potential microscopy (SSPM)^23^. Using SSPM, Kalinin and Bonnell could observe contrast above T_C_ which decreases with time. They attributed this phenomenon to the desorption of adsorbates. Adsorbates could pin the FE distortion, as, for example, at the surface of BaTiO_3_ single crystals^26^ and thin films^23,48^. In fact, complete desorption of dissociated water from the BaTiO_3_ surface only occurs at higher temperatures, typically around 675 K^48^. Residual adsorbates might contribute to the persistence of domain related contrast in the paraelectric phase in their case.

The ultra-high vacuum conditions in our experiment makes this interpretation unlikely. In particular, XPS spectra did not show any particular contamination. The self-reversal of the dagger domains is not related to contamination as it happens as a quick jump rather than a slow transport phenomenon^23^. In fact, we observe two self-reversal processes. The first occurs at 373 K and is the self reversal of the tweed in the ferroelectric state. This is consistent with the capacity of tweed to take on different polarization directions and we suggest that the reversal happens at temperature which unpins the tweed polarization. The second polarization reversal occurs well above T_C_, greater than 500 K and represents reversal of the surface domain polarization to compensate the increase in the depolarizing field.

In terms of the temperature range our results are similar to those of Höfer *et al*. who observed domain patterns up to 510 K and self-reversal of the surface polarization. Above this temperature their sample surface was uniform in PEEM. We see a higher contrast between the various domain states with an abundance of finer tweed structure and can follow this contrast to a higher temperature (550 K). Höfer *et al*. ascribe the above T_C_ contrast to tetragonality favored by the outward movement of oxygen anions. To simulate this they use a bulk tetragonal structure and relax the surface layers. This rumpling effect is very common for ABO_3_ surfaces including non-ferroelectric materials SrTiO_3_, CaTiO_3_ or SrZrO_3_^49^. Surface tetragonality above T_C_ is likely because of the natural tendency for anions to move upwards. We suggest that it persists despite the bulk transition into the cubic structure and is in fact stabilized thanks to the existence of dagger domains above the invariant plane rather than being due to an underlying tetragonal structure.

The polarization charges at the surface can be assumed to be screened over a so-called dead layer. Within the framework of this model, the work function difference is directly proportional to the surface polarization^50^. For inwards and outwards pointing polarization, the work function difference can be written as 2 (e/*ε*_0_) *P*_*R*_*d*, where e is the electronic charge, *ε*_0_ the permittivity of free space, *P*_R_ the polarization and d an effective distance rumpling. The work function difference above T_C_ between domains with up and down polarization is 0.24 eV. Assuming a typical d value of 0.1 Å gives 10.6 *μ*C/cm^2^ for the surface polarization charge, compared with 26 *μ*C/cm^2^ for bulk BaTiO_3_.

Note that dipolar defects (resulting from oxygen vacancies) can also contribute to imprint the polar tetragonality. Interestingly, the surface keeps the fingerprint of the bulk ferroelectric state and the surface memory loss occurs in our experiments at a higher temperature (≥550 K). The transition temperature for the memory loss of the FE domain structure (without considering desorption)^48^ occurs within the cubic phase ranges between 506 K and 586 K in the literature^15,17,24^.

Equally, our results are consistent with the phenomenon of self-reversal, although in our case it occurs at higher temperatures inside the stability range of the cubic bulk phase rather than just above T_C_. The main difference is the observation and characterization of tweed, which was not reported by Höfer *et al*., probably because of the more than twice bigger FoV (150 *μ*m against 67 *μ*m in our case). Indeed closer inspection of their images reveals a similarly mottled texture, which may well be related to our tweed pattern. In addition, recent first-principles calculations in conjunction with far-infrared measurements^51^ have demonstrated that two different overdamped modes contribute to the dielectric response of BaTiO_3_ cubic phase and both modes show an inflection in their temperature dependence at about 550 K. The second mode (at ~70 cm^−1^ close to T_C_) shows the strongest inflection and has been associated to small correlated regions of needle-like shape. These results can be fully connected with our results above for the ferroelectric surface.

In conclusion, we have used photoemission electron microscopy to study the evolution of surface charges through the FE to PE phase transition in BaTiO_3_ (001) and deep into the PE bulk phase. At room temperature, the surface displays arrays of ferroelectric domains superimposed with a clear signature of tweed. The imprint of the initial contrast remains visible well above the bulk transition temperature, up to 550 K. The domain patterns above T_C_ are therefore surface proximity effects. They represent exactly the same patterns as in the ferroelectric phase and are hence a robust fingerprint of the domain arrangement of the bulk FE state which can conserve the domain information up to 150 K above T_C_. At lower temperatures (below 550 K), the surface domain structure is pinned, presumably by a combination of point defects and the quasi-static tweed. Two polarization self-reversals occur, first the tweed below T_C_, then the domain polarization above T_C_. Both reversals minimize the depolarizing field at the surface. The FE order changes after annealing at high temperatures (975 K), implying that both the tweed and the domain structure are unpinned. The new pattern at room temperature is again fully compatible with the FE state of the bulk and microscopic surface fluctuations of the order parameter, leading to tweed.

## Methods

### Photoemission electron microscopy

The sample is a BaTiO_3_ (001) single crystal, supplied by SurfaceNet GmbH. Before insertion into the vacuum system, the sample was exposed for 5 minutes to ozone using a UV lamp in air to remove surface organic contamination. It was then inserted into a dry load lock and pumped down within one minute. The sample was then annealed in ultra-high vacuum (UHV) several times for 30 minutes at temperatures between 975 and 1025 K until no residual charging of the surface was observed in PEEM. UHV annealing creates oxygen vacancies to increase surface conductivity and minimize charging during the PEEM experiments^52^. The surface cleanliness was checked by X-ray photoemission spectroscopy (XPS) and its crystallinity by low energy electron diffraction (see Supplementary Materials S4). The temperature was measured with a Pt100 resistor (4-wire connection) which is located next to the heater and the sample plate. The base pressure for all measurements was in the low 10^−10^ mbar range.

The PEEM is a NanoESCA (ScientaOmicron) and comprises a fully electrostatic PEEM column, followed by an imaging double energy analyzer, as an energy filter^53^. The PEEM images were acquired using a He lamp (21.2 eV) and a 67 *μ*m field of view (FoV). The spatial resolution is 50 nm and the overall energy resolution was 0.2 eV to allow rapid image acquisition during heating and cooling cycles. The photoelectron kinetic energy (E) is measured with respect to the Fermi level (E_F_) of the sample holder. Thus, for a metallic sample, the work function is the value of E-E_F_ for the photoemission threshold. Note that the high extractor voltage reduces the threshold value via the Schottky effect^54^, in this case by 136 meV. The image series as a function of energy are corrected for non-isochromaticity due to vertical dispersive plane^55^. They directly measure the variation in the local work function (i.e. the photoionization potential in the case of a material with non-zero band gap), in the 67 *μ*m FoV. A quantitative analysis is performed using the photoemission spectrum extracted from each pixel, fitted with a complementary error function. The procedure generates a 2D map of the work function, corresponding to the energy position of the threshold^56^.

## Electronic supplementary material

S3 PEEM movie

Supplementary materials

## Data Availability

The datasets generated during and/or analysed during the current study are available from the corresponding author on reasonable request.
