# Supplementary material for: Surface Proximity Effect, Imprint Memory of Ferroelectric Twins, and Tweed in the Paraelectric Phase of BaTiO3
Source: Sci Rep. 2018 Sep 12;8:13660. doi: 10.1038/s41598-018-31930-4 (PMC6135802; doi:10.1038/s41598-018-31930-4)
Supplement: Supplementary file 2 — Supplementary materials [file 41598_2018_31930_MOESM2_ESM.docx]

Supplementary materials

**Surface Proximity Effect, Imprint Memory of Ferroelectric Twins, and Tweed in**

**the Paraelectric Phase of BaTiO_3_**

C. Mathieu^1^, C. Lubin^1^, G. Le Doueff^1^, M. Cattelan^2^, P. Gemeiner^3^, B. Dkhil^3^, E. K. H. Salje^4^, N. Barrett^1^

*S1. Determination of the Curie temperature in situ using PEEM*

At T_C_, the BatiO_3_ undergo a phase transition from a tetragonal to a cubic phase. Therefore, in the cubic phase, where the ferroelectricity is lost, no surface charges are expected anymore. Hence, the contrast in PEEM should disappear above T_C_. However, this is not the case, as explained in the paper. This remaining contrast above T_C_ prevents us to easily observe the phase transition while measuring in PEEM. Another way to determine the Curie temperature is by using the mechanical deformation of the material. While going through the tetragonal to the cubic phase, the c-parameter will shrink while the a-parameter will slightly expand. In the case of a single crystal with a unique polarization state, 0.1% change in the lattice parameter will induce a 10 µm displacement. BaTiO_3_ single crystals naturally present different polarization states; thus the total displacement is expected to be smaller. Figure S1 presents PEEM images below and above T_C_. An isolated defect is used as a marker to track the movement of the crystal surface through the transition. In this example, the defect move of 6.6 µm at the transition, easily observable in PEEM.


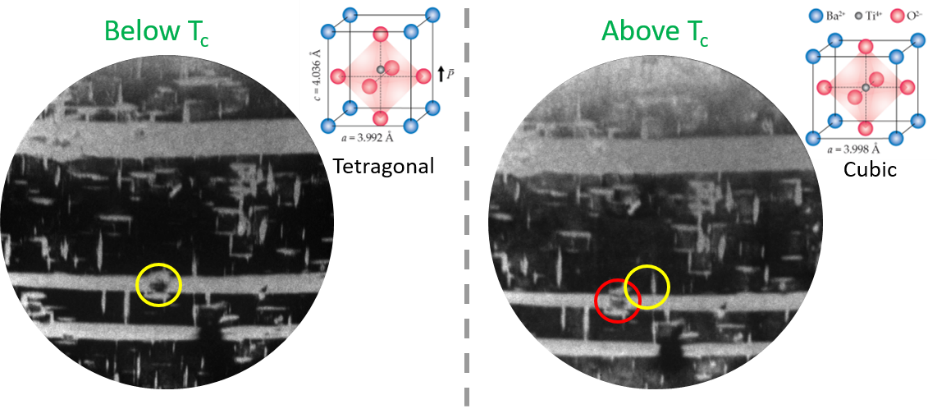


***Figure S1****: PEEM images below and above Tc. The defect is used as a marker to follow the mechanical deformation. The yellow (red) circle represents the position of the defect below (above) the phase transition. The field of view is 67 µm.*

*S2. Raman spectroscopy measurements*

Raman spectroscopy measurements were performed using a LABRAM Horiba Raman spectrometer in the range 50 cm^−1^ to 800 cm^−1^ using 632.8 nm HeNe laser as excitation source allowing thus to probe the same BTO sample down to 500-600 nm thick below the surface. The spectra were measured under ambient pressure conditions, but in the same temperature range used in PEEM, i.e. from 300 K to 550 K. The temperature evolution of the Raman phonon spectrum near 310 cm^-1^ and 700 cm^-1^ corresponding to the vibration modes assigned as E(LO+TO)+B_1_ and E(LO)+A_1_(LO), respectively, is shown in Fig.S3. E(TO) mode at 310 cm^-1^ corresponds to the mode characteristic of the long range polar tetragonal order involving Ti^4+^ displacement while the 700 cm^-1^ modes correspond to oxygen octahedra vibrational response. The Raman lines typical of the ferroelectric distortions disappear in the paraelectric phase for symmetry reasons. The intensity collapse at the transition point T_C_ (=393K) is complete, no residual Raman lines were observed at higher temperatures. This proves that the stepwise transition follows the classic Landau-Ginzburg type behavior without any indication for ferroelectric domain patterns in the cubic phase [Salje1983, Salje1997].

***Figure S2****: Raman spectrum of the BaTiO_3_, as a function of the temperature in steps of 10 K from 300 to 550 K.*

*S3. Ferroelectric domain self-reversal*

**Figure S3.** Movie of the self-reversal polarization in the surface layer.

The movie presented in Fig. S3 is recorded at a fixed energy (E-E_F_=4 eV) while increasing the temperature. The temperature range of the movie is above Tc, from 450 to 550 K. This experiment has been performed after an annealing at 975 K, and is related to the results presented figure 3. There are horizontal orientations of the polarization, along with tweeds. During the movie, the sample position changes due to a thermal drift. Below 510 K, the thin horizontal domains are brighter than the larger domains. This contrast is reversed around and above 515 K, where the backswitching of the domains occur.

*S4. Surface characterization*

To avoid charging, the BTO sample was annealed several times. X-ray photoemission spectroscopy was performed before and after each annealing. All the spectra were similar. The XPS survey, recorded before the presented results in the article, is presented Fig. S2 (a). It shows all of the principal core level peaks associated to BaTiO_3_. There is no or very low (less than 1%) surface contamination, usually highlighted by the presence of a carbon peak around 284 eV. The surface is therefore clean from all surface contaminations.

Figure S2 (b) presents the low energy electron diffraction (LEED) pattern, obtained for an energy of 100 eV. The sharp (1×1) pattern highlights the well-reconstructed surface of the BTO **and allows determination of the crystal axes in the PEEM images.**


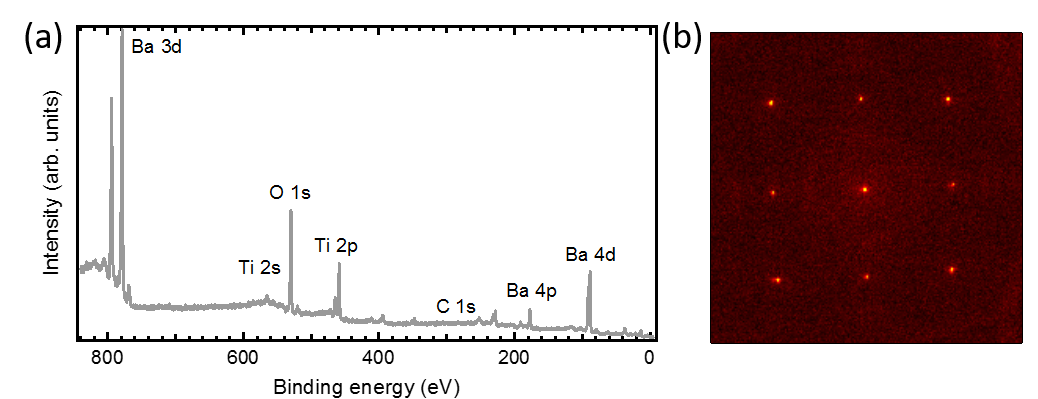


***Figure S4.*** *(a) Area-averaged X-ray photoelectron spectroscopy survey spectrum recorded using Al Kα X-ray source (hν=1486.7 eV). (b) LEED pattern, obtained at 100 eV.*

***Figure S5.*** *(a) Ti 2p, (b) Ba 4d and (c) C 1s XPS spectra before (lower, blue curves) and after (upper, red curves) annealing at 975 K.*

**Figure S5 shows the Ti 2p, Ba 4d and C 1s XPS spectra before (lower, blue curves) and after (upper, red curves) annealing at 975 K. The Ti 2p spectrum shows no evidence for an increase in the oxygen vacancy concentration after annealing at 975 K supporting the hypothesis of unpinning at this temperature. This is consistent with typical oxygen vacancy to domain wall binding energy of 100-250 meV [He2003]. However, a quantitative analysis of the absolute vacancy concentration would require a higher spectral resolution than that available in the present set-up and will be the subject of a future dedicated study. The C 1s spectra in Fig. S5c are identical before and after 975 K annealing, therefore the loss of ferroelectric memory at the surface is not correlated with the level of carbon contamination which, as can be seen is in any case extremely low.**

**References**

[Salje1983] E. K. H. Salje, V. Devarajan , U. Bismayer, D. M. C. Guimaraes, [*J. Phys. C Solid State*, **16**, 5233 (1983)](http://iopscience.iop.org/article/10.1088/0022-3719/16/27/008).

[Salje1997] E. K. H. Salje, U. Bismayer, [*Phase Transit.*, **63**, 1 (1997)](https://www.tandfonline.com/doi/abs/10.1080/01411599708228789).

[He2003] L. He and D. Vanderbilt, Phys. Rev. B 68, 134103 (2003).
